# Preclinical evaluation of immunogenicity and protective efficacy of a recombinant chimeric protein vaccine against visceral leishmaniasis

**DOI:** 10.1017/S0031182024001240

**Published:** 2025-07

**Authors:** Daniela P. Lage, Danniele L. Vale, Fabiana A. G. Maia, Vívian T. Martins, Marcela G. P. Silva, Nathalia C. Galvani, Mariana M. Cardoso, Gabriel J. L. Moreira, Eduarda M. Sombrio, Camila S. Freitas, Breno L. Pimenta, Karolina O. M. Falcão, Saulo S. G. Dias, Raquel S. Bandeira, Isabela A. G. Pereira, Grasiele S. V. Tavares, Antônio L. Teixeira, Miguel A. Chávez-Fumagalli, Bruno M. Roatt, Ricardo A. Machado-de-Ávila, Eduardo A. F. Coelho

**Affiliations:** 1 Programa de Pós-Graduação em Ciências da Saúde: Infectologia e Medicina Tropical, Faculdade de Medicina, Universidade Federal de Minas Gerais, Belo Horizonte, Minas Gerais, Brazil; 2 Laboratório de Imunopatologia, Núcleo de Pesquisas em Ciências Biológicas, Departamento de Ciências Biológicas, Insituto de Ciências Exatas e Biológicas, Universidade Federal de Ouro Preto, Ouro Preto, Minas Gerais, Brazil; 3 Programa de Pós-Graduação em Ciências da Saúde, Universidade do Extremo Sul Catarinense, Criciúma, Santa Catarina, Brazil; 4 Departamento de Patologia Clínica, COLTEC, Universidade Federal de Minas Gerais, Belo Horizonte, Minas Gerais, Brazil; 5 Neuropsychiatry Program, Department of Psychiatry and Behavioral Sciences, McGovern Medical School, The University of Texas Health Science Center at Houston, Houston, TX, USA; 6 Computational Biology and Chemistry Research Group, Vicerrectorado de Investigación, Universidad Católica de Santa María, Urb. San José S/N, Umacollo, Arequipa, Peru

**Keywords:** chimera, immune response, lymphoproliferation, T-cell epitopes, vaccine, visceral leishmaniasis

## Abstract

Visceral leishmaniasis (VL) is a tropical disease that can be fatal if acute and untreated. Diagnosis is difficult, the treatment is toxic and prophylactic vaccines do not exist. *Leishmania* parasites express hundreds of proteins and several of them are relevant for the host's immune system. In this context, in the present study, 10 specific T-cell epitopes from 5 parasite proteins, which were identified by antibodies in VL patients’ sera, were selected and used to construct a gene codifying the new chimeric protein called rCHI. The rCHI vaccine was developed and thoroughly evaluated for its potential effectiveness against *Leishmania infantum* infection. We used monophosphoryl lipid A (MPLA) and polymeric micelles (Mic) as adjuvant and/or delivery system. The results demonstrated that both rCHI/MPLA and rCHI/Mic significantly stimulate an antileishmanial Th1-type cellular response, with higher production of IFN-*γ*, TNF-*α*, IL-12 and nitrite in vaccinated animals, and this response was sustained after challenge. In addition, these mice significantly reduced the parasitism in internal organs and increased the production of IgG2a isotype antibodies. *In vivo* and *in vitro* toxicity showed that rCHI is safe for the mammalians, and the recombinant protein also induced *in vitro* lymphoproliferative response and production of Th1-type cytokines by human cells, which were collected from healthy subjects and treated VL patients. These data suggest rCHI plus MPLA or micelles could be considered as a vaccine candidate against VL.

## Graphical abstract

**Figure:**
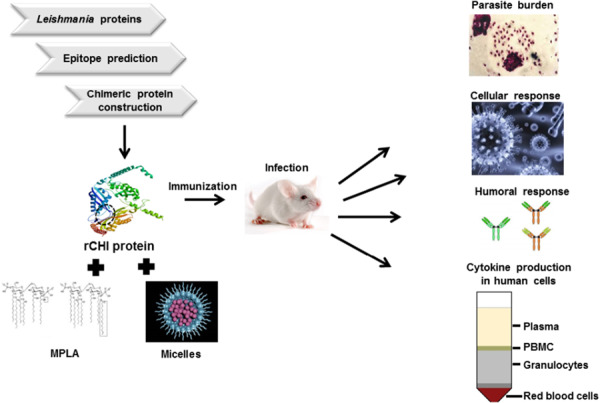

## Introduction

Leishmaniases are parasitic diseases responsible for causing a broad clinical spectrum in patients, with the main forms being known as tegumentary leishmaniasis (TL) and visceral leishmaniasis (VL). These diseases are endemic in 98 countries and territories around the world. Approximately 1 billion people live in endemic areas, and it is estimated that 1 million new cases of TL and 30 000 new cases of VL occur annually (WHO, 2024). VL is the most severe form of leishmaniasis, which can be fatal if acute and untreated, and is caused by *Leishmania donovani* and *L. infantum* species. The development of active disease depends on the factors such as the immune status and nutritional condition of the patients, and virulence of infective parasite strain (Burza *et al*., 2018; Montaner-Angoiti and Llobat, 2023).

Public health measures to prevent VL are mainly based on vector control, patient detection and treatment and reservoir elimination (Brito *et al*., 2024). However, drugs used for the VL treatment present limitations, such as organic toxicity, high cost and the development of resistant strains (Akbari *et al*., 2021). Control of vector-borne diseases is challenging due to the erratic nature of these insects (Balaska *et al*., 2021), and the diagnosis of disease is hampered by varying sensitivity and specificity of laboratorial tests (Erber *et al*., 2022). Considering the impact of the disease on public health and the need to develop more effective control strategies, the development of vaccines could be a promising approach, since they can induce a lasting effect, and be administered with prophylactic and therapeutic purposes in patients (Kumari *et al*., 2023). Although attempts have been made to obtain effective vaccine candidates to protect against VL (He *et al*., 2019; Emerick *et al*., 2021; Yadav *et al*., 2021; Kushwaha and Capalash, 2023; Ojha *et al*., 2023), few canine vaccines are available and present contradictory results due to distinct factors; and a human vaccine does not exist (Srivastava *et al*., 2016; Le Rutte *et al*., 2020).

In the last decades, several *Leishmania* antigens were evaluated as vaccine candidates administered as recombinant proteins, synthetic peptides, DNA-based plasmids, among others, with satisfactory protective response obtained in experimental models (Fiuza *et al*., 2015; Viana *et al*., 2016; Abdellahi *et al*., 2022; Tandon *et al*., 2023; Avishek *et al*., 2024). One strategy employed by researchers to elicit strong host immune response is the identification of protein epitopes that bind to major histocompatibility complex (MHC) molecules (Germanó *et al*., 2022). It is known that proteins possess immunodominant T-cell epitopes, which induce immunity through presentation via MHC on the surface of antigen-presenting cells (APCs) (Basmenj *et al*., 2023). Thus, identifying the target epitopes can be efficient for the development of effective multi-epitope vaccines (Kumar *et al*., 2023). In addition, the advancement of the bioinformatics has made it easier to identify potential T-cell epitopes of immunogenic candidates, such as those specific to human and murine cells antigens. Recombinant multi-epitope vaccines have greater potential for mass application, since they can express many immunogenic parts of proteins simultaneously (Clímaco *et al*., 2023). This becomes especially challenging when considering the complex nature of VL and the genetic polymorphism of the host immune system (Coler *et al*., 2015). These new chimeric proteins have other advantages, such as the absence of infectious materials in their structural nature, good specificity and the potential for large-scale production at a lower cost (Ostolin *et al*., 2021; Lage *et al*., 2022; Ojha *et al*., 2023). Therefore, immunogenic peptides can be identified within proteins previously described as vaccine candidates.

This work identified immunogenic T-cell epitopes from 5 *L. infantum* proteins [ATP synthase subunit beta mitochondria (SUZ42449.1), dihydrolipoamide dehydrogenase (AYU81901.1), dihydrolipoamide acetyltransferase precursor (SUZ46653.1), S-adenosylhomocysteine hydrolase (AYU83765.1) and vacuolar proton pump subunit B (AYU80416.1)], which were recently identified by antibodies in VL patient sera by an immunoproteomics approach performed by Machado *et al*. (2022a), and used them to construct the gene codifying a new chimeric protein called rCHI. Ten immunogenic epitopes specific to human and murine CD4^+^ and CD8^+^ T cells were identified and used to construct the chimera. This chimera was then produced and tested in BALB/c mice against *L. infantum* infection, being associated with monophosphoryl lipid A (MPLA) and polymeric micelles (Mic) as an adjuvant and/or delivery system. Results showed that both combinations induced immunogenicity before and after infection, leading to significant reductions in the parasite burden in the infected animals. The animals developed a specific Th1-type cellular and humoral response, suggesting that rCHI plus MPLA or micelles could be considered as vaccine candidates to protect against VL.

## Materials and methods

### Reagents

A list containing the description of the antibodies and commercial kits used in this study is presented, with information about their catalogue numbers, source, company name, clone, among others (Supplementary Table 1).

### Chimera design and epitope selection

For the construction of gene codifying rCHI, amino acid sequences from 5 parasite proteins were subjected to bioinformatics assays to select regions specific to murine and human T cell haplotypes containing immunogenic epitopes. For this, prediction of CD4^+^ and CD8^+^ T cells epitopes was performed using the Rankpep program (Reche and Reinherz, 2007). The FASTA sequence of each protein was added to the input field and the alleles A02.01, A03.01, A24 and B07.02 were selected for human CD8^+^ T (MHC-I) cells, while the alleles H-2Db, H-2Dd, H-2Kb, H-2Kd, H -2Kk and H-2Ld were selected for murine CD8^+^ T cells. In the same way, the alleles HLA-DR1, HLA-DR2, HLA-DR3, HLA-DR4, HLA-DR5, HLA-DR8 (DRB1*0801), HLA-DR9, HLA-DR11 (DRB1*1101), HLA-DR12 (DRB1*1201), HLA-DR13 (DRB1*1301) and HLA-DR15 (DRB1*1501) were selected for human CD4^+^ (MHC-II) T cells, while the alleles H-2IAb, H-2IAd, H-2IAs, H-2IEd and H-2IEb were selected for murine CD4^+^ T cells. Then, for the ‘Binding threshold’ parameter, 2% was used for MHC-I and 5% for MHC-II, and in ‘Proteasome cleavage’ filter off was used. Next, we clicked send and the selected sequences were those that presented the highest percentage of prediction. The ABCpred program was used to predict B-cell epitopes, with a threshold of 0.85 in at least Window length used for the prediction of 16 (Saha and Raghava, 2006). As a consequence, protein regions containing specific T-cell epitopes from humans and mice, but without B-cell epitopes, were selected to construct the gene codifying rCHI. The ProtParam server (Gasteiger *et al*., 2005) was used to predict the physical and chemical characteristics of protein including molecular weight, isoelectric point, amino acid residues, estimated half-life, instability index, aliphatic index and grand average of hydropathicity (GRAVY). The PepCalc server (www.pepcalc.com) was used to predict the protein solubility. The prediction of the secondary structure was performed by Jpred v.4 program.

### Expression and purification of rCHI protein

The gene encoding rCHI was commercially synthesized by GenScript^®^ (catalogue 1586730; Piscataway, NJ, USA). It was cloned into a pET-28a(+) expression vector and transformed into Artic Express cells (DE3, Agilent Technologies, USA). The rCHI expression was induced after the addition of 0.5 mm isopropyl *β*-D-1-thiogalactopyranoside (Sigma-Aldrich, St. Louis, MO, USA), and cultures were shaken at 200 × *g* per min for 24 h at 12°C. Cells were ruptured by ultrasonication with 6 cycles of 30 s at 38 MHz, followed by 6 cycles of freezing (−196°C) and thawing (+37°C). Debris was removed by centrifugation and rCHI was purified using HisTrap HP affinity column connected to an AKTA system (GE Healthcare Life Sciences, USA). The eluted fractions containing the protein were concentrated using Amicon^®^ ultra15 centrifugal filters 10 000 NMWL (Millipore, Germany) and purified on a Superdex™ 200 gel-filtration column (GE Healthcare Life Sciences). After purification, rCHI was passed through a polymyxin-agarose column to remove the residual endotoxin content (<10 ng of lipopolysaccharide per 1 mg of protein) using the Pierce™ Chromogenic Endotoxin Quant Kit (Thermo Scientific, Waltham, MA, USA).

### Preparation of the protein-containing micelles

To prepare rCHI-containing micelles, Poloxamer P407 (18% w/w; Sigma-Aldrich) was added to 1 ×  phosphate-buffered saline (PBS) pH 7.4 with moderate magnetic agitation and on ice bath. Complete dissolution of the polymer occurred after incubation for 18 h at 4°C. The protein (1.0 mg) was added to a microtube containing 1% (v/v) dimethyl sulfoxide, which was used as a co-solvent and solubilized using vortex mixing. It was added immediately to the Poloxamer P407 until a viscous emulsion was obtained. The Poloxamer P407 was kept at 4°C, and the mixture was freshly prepared on the day of immunization. The protein concentration was estimated at 0.79 mg mL^−1^ as described by (Pellosi *et al*., 2016). Empty micelles (18% w/w) were prepared using the same protocol, but without addition of rCHI.

### Mice, parasite and preparation of *Leishmania* antigenic extract

Female BALB/c mice of 8 weeks of age were maintained under pathogen-free conditions. *Leishmania infantum* (MHOM/BR/1974/PP75) promastigotes were cultured at 24°C in complete Schneider's medium (Sigma-Aldrich), which was supplemented with 20% (*v*/*v*) heat-inactivated fetal bovine serum (FBS; Sigma-Aldrich), 20 mm L-glutamine, 200 U mL^−1^ penicillin and 100 μg mL^−1^ streptomycin (Sigma-Aldrich) at pH 7.4 (Coelho *et al*., 2003). Soluble *Leishmania* antigenic extract (SLA) was prepared using 10^9^ stationary-phase promastigotes as described by Coelho *et al*. (2003).

### Vaccination and challenge infection

Mice (*n* = 16 per group) received the following treatment subcutaneously in their left hind footpad: (i) 50 μL of PBS (saline group); (ii) 50 μL of micelles (5 mg kg^−1^ body weight; Mic group); (iii) 50 μL of MPLA (25 μg; catalogue 1246298-63-4, Sigma-Aldrich) (MPLA group); (iv) 50 μL of chimera (25 μg) (rCHI group); (v) 50 μL of chimera (25 μg) plus MPLA (25 μg) (rCHI/MPLA group); and (vi) 50 μL of chimera incorporated into polymeric micelles (25 μg of protein and 5 mg kg^−1^ body weight of micelle) (rCHI/Mic group). Three doses were administered at 15-day intervals. Thirty days after the last dose, mice (*n* = 8 per group) were euthanized, and blood samples and spleens were collected for immunological assays. The other animals (*n* = 8 per group) were subcutaneously infected in their right hind footpad with 10^7^ *L. infantum* stationary promastigotes. These animals were followed by 45 days, when they were euthanized and blood samples, spleens, livers, bone marrows (BMs) and draining lymph nodes (dLNs) were collected to perform the immunological, biochemical and parasitological analyses. The experimental strategy adopted in this study was based on others, recently described by Dias *et al*. (2018) and Lage *et al*. (2020, 2022).

### Cytokine and nitrite production

Cytokine response and nitrite production were measured as described by Lage *et al*. (2022). Briefly, animals’ spleens (*n* = 8 mice per group, each step) were collected before or after challenge, and splenocytes (5 × 10^6^ cells per well) were incubated in DMEM added with 20% FBS, 20 mm L-glutamine, 200 U mL^−1^ penicillin and 100 μg mL^−1^ streptomycin at pH 7.4. Cells were either left unstimulated (background, control) or stimulated with rCHI or SLA (10 and 25 μg mL^−1^, respectively) for 48 h at 37°C in 5% CO_2_ incubator. IFN-*γ*, IL-4, IL-10 and IL-12 levels were measured in culture supernatants by capture ELISA (BD OptEIA TM set mouse kits, Pharmingen^®^, San Diego, CA, USA), as per the manufacturer's protocol. Cell culture supernatant prepared after infection was also used to nitrite quantify through the Griess reaction. For this, 50 μL of culture supernatant was mixed with an equal volume of reagent, and absorbances were measured at 540 nm. The concentration of nitrite was calculated from a standard curve.

### IFN-*γ* mRNA expression in SLA-stimulated cell cultures

A reverse transcription-quantitative polymerase chain reaction (RT-qPCR) assay was developed to evaluate the IFN-*γ* mRNA expression in stimulated spleen cell cultures as described by Lage *et al*. (2023). For this, spleens were removed from euthanized animals (*n* = 8 per group, each step) collected before and after challenge, and RNA was extracted using TRIzol reagent (Invitrogen, Carlsbad, CA, USA) as per the manufacturer's protocol. RNA was resuspended in UltraPure™ DNase/RNase-free distilled water (Invitrogen) and concentration was measured spectrophotometrically in a NanoDrop LITE reader (Thermo Scientific), with absorbance ratios of 260/280 nm. RNA quality was evaluated in an electrophoresis in 1.5% agarose gel, and it was treated for 15 min at room temperature with DNAse (Invitrogen). This enzyme was deactivated using 25 mm EDTA for 10 min at 65°C, and RNA (2 μg) was reverse transcribed in an Applied Biosystems High-Capacity cDNA Reverse Transcription kit (Thermo Scientific), forming cDNA using the following parameters: 25°C for 10 min, 37°C for 120 min and 85°C for 5 min. RT-qPCR was performed using an Applied Biosystems PowerUp™ SYBR™ Green PCR master mix (Thermo Scientific), with gene-specific primers for IFN-*γ*: 5′-TCAAGTGGCATAGATGTGGAAGAA-3′ (*forward*) and 5′-TGGCTCTGCAGGATTTTCATG-3′ (*reverse*), in a 7900HT Thermocycler (Applied Biosystems). Transcripts were normalized using ACTB and GAPDH housekeeping genes. The procedure was optimized by adjusting the primer concentrations to 5.0, 10.0 and 15.0 pmol to test for optimal specificity and efficiency. The purity of PCR products was evaluated through melting curves and gel electrophoresis. The cycle parameters were: (i) initial denaturation at 95°C for 10 min, (ii) 40 cycles at 95°C for 15 s, (iii) annealing/extension at 60°C for 1 min and (iv) dissociation stage for recording the melting curve. Results are shown graphically as the fold changes in gene expression by using the mean ± standard deviation of target gene. Data were analysed by the relative expression using the 2^−ΔΔCT^ method.

### Flow cytometry

A flow cytometry was performed to evaluate the frequency of T cells producing IFN-*γ*, TNF-*α* and IL-10 in SLA-stimulated cell cultures, which were collected after infection as described by Brito *et al*. (2020). Spleen cells (5 × 10^6^ per well) were incubated in complete RPMI medium in 96-well round-bottom plates, and were either left unstimulated (medium) or stimulated with SLA (25 μg mL^−1^) for 48 h at 37°C in 5% CO_2_ incubator. Brefeldin A (Sigma-Aldrich) (10 mg mL^−1^) was added in the cultures, 4 h before the end of the period of incubation. Some cultures were stimulated with Phorbol 12-myristate 13-acetate (5 ng mL^−1^) and ionomycin (1 mg mL^−1^), used as positive control. Subsequently, cells were stained by Fixable Viability Stain 450 (FVS450, BD Biosciences, San Diego, California, USA) for 15 min at room temperature, followed by labelling with antibodies against CD3^+^ (BV650 anti-mouse, clone 145.2C11), CD4^+^ (BV605 anti-mouse, clone RM4-5) and CD8^+^ (BV786 anti-mouse, clone 53–6.7) for 30 min at room temperature. Cells were fixed with FACS fixing solution, washed and permeabilized with PBS buffer plus 0.5% saponin and stained with antibodies against IFN-*γ* (AF700 anti-mouse, clone XMG1.2), TNF-*α* (PE-Cy7 anti-mouse, clone LG.3A10) and IL-10 (APC anti-mouse, clone JES5-16E) for 30 min at room temperature. All antibodies were purchased from BD Biosciences. Cells acquisition (100 000 events) was performed on LSR Fortessa cytometer (BD Biosciences) using FACSDiva software. FlowJo software was used for analysis, dead cells were excluded after FVS450 stain and alive cells were gated for stained CD3^+^CD4^+^ and CD3^+^CD8^+^ T cells and intracellular cytokine production. The frequencies of positive T cells were evaluated in terms of percentages, in which ratios between the SLA-stimulated (SC) *vs* unstimulated (medium, CC) cultures were calculated and showed as indexes. The gating strategy used to characterize the frequency (in percentage) of T cells producing the cytokines IFN-*γ*, TNF and IL-10 through the Boolean gate strategy is shown (Supplementary Fig. 1).

### Analysis of humoral response

The antibody production was evaluated in animals’ sera, which were collected before and after infection (*n* = 8 per group, each step). Levels of anti-rCHI and anti-SLA IgG1 and IgG2a isotypes were measured as described by Lage *et al*. (2022). Previous titration curves were established to obtain the most appropriate concentration of antigen and serum dilutions to be used in the assays. Microtiter plates (Jetbiofil^®^, Belo Horizonte, Minas Gerais, Brazil) were coated with rCHI or SLA (0.25 and 1.0 μg per well, respectively) in coating buffer (50 mm carbonate buffer, pH 9.6) for 16 h at 4°C. Free binding sites were blocked using 250 μL of PBS-T (PBS plus Tween 20 0.05% *v*/*v*) added with 5% (*w*/*v*) bovine serum albumin for 1 h at 37°C. After washing the plates 5 times with PBS-T, wells were incubated with 100 μL of individual sera (1:100 diluted in PBS-T) for 1 h at 37°C. Plates were washed 5 times with PBS-T and incubated with antibodies specific to IgG1 (1: 10 000 diluted in PBS-T; rat anti-mouse IgG1 secondary antibody, peroxidase-conjugated antibody, catalogue SA1-35640, Invitrogen) or IgG2a (1: 20 000 diluted in PBS-T; rat anti-mouse IgG2a secondary antibody, peroxidase-conjugated antibody, catalogue SA1-35646, Invitrogen) isotypes. After incubation for 1 h at 37°C, wells were washed 5 times with PBS-T, and reactions were developed using H_2_O_2_, ortho-phenylenediamine and citrate–phosphate buffer pH 5.0, for 30 min and in the dark. Reactions were stopped by adding 2N H_2_SO_4_, and optical density (OD) values were read in a spectrophotometer (Molecular Devices, Spectra Max Plus, Concord, Canada) at 492 nm.

### Organ toxicity

Sera samples were collected from mice (*n* = 8 per group) 45 days after challenge, when levels of alanine transaminase (ALT), aspartate transaminase, urea and creatinine enzymes were measured as described by Machado *et al*. (2022b). The evaluations were performed using commercial kits (Labtest Diagnostica^®^, Belo Horizonte, Minas Gerais, Brazil) as per the manufacturer's protocol. Sera from non-infected and non-immunized mice (*n* = 5) were used as control.

### Parasite load evaluated through a limiting dilution assay

The parasite load was evaluated in the infected animals (*n* = 8 per group) as described by Freitas *et al*. (2023). Spleens, livers, BMs and dLNs were collected and macerated in a glass tissue grinder using sterile PBS, and tissue debris was removed by centrifugation at 150 × *g*. Cells were concentrated by centrifugation at 2000 × *g* and resuspended in 1 mL of complete Schneider's medium, and serially diluted 10^−1^ to 10^−12^ in the same medium. Each sample was plated in triplicate and cultured for 7 days at 24°C. Results were expressed as the negative log of the titre (the dilution corresponding to the last positive well) adjusted per milligram of organ.

### Parasitism evaluated by quantitative polymerase chain reaction (qPCR)

Splenic parasitism was also evaluated by qPCR after infection (*n* = 8 per group) as described by Machado *et al*. (2022a). DNA content was extracted using Wizard Genomic DNA Purification Kit (Promega Corporation, Madison, WI, USA), and suspended in UltraPure™ DNase/RNase-free distilled water (Invitrogen). Parasite load was estimated using primers to amplify *L. infantum* kDNA: *forward* 5’-CCTATTTTACACCAACCCCCAGT-3’ and *reverse* 5’-GGGTAGGGGCGTTCTGCGAAA-3’. These primers are used to amplify a *Leishmania* specific and conserved region, which is used as an indicative of the presence of parasite DNA. Primers (*forward* 5’-CAGAGCAAGAGAGGTATCC-3’ and *reverse* 5’-TCATTGTAGAAGGTGTGGTGC-3’) were used to amplify the mouse *β*-actin gene, as endogenous control. Reactions were developed in a 7500 HT apparatus (96-well plate; Applied Biosystems) using the Applied Biosystems PowerUp™ SYBR™ Green PCR master mix (5 μL; Thermo Fisher, Waltham, MA, USA) added with 2 mm of each primer (1 μL) and 4 μL of DNA (25 ng μL^−1^). Samples were incubated for 10 min at 95°C and submitted to 40 cycles of 15 s at 95°C and 1 min at 60°C. At each time point, fluorescence data were collected and results were calculated by interpolation from a standard curve included in the same run, which was prepared in duplicate and expressed as the number of *L. infantum* organisms per total DNA.

### Human blood samples and cytokine production

Blood samples (20 mL) were collected from VL patients (*n* = 6, including 2 males and 4 females, with ages ranging from 27 to 54 years), before and 6 months after their treatment, which was performed with pentavalent antimonials (Sanofi Aventis Farmacêutica Ltda., Suzano, São Paulo, Brazil). In addition, blood samples were also collected from healthy individuals (*n* = 6, including 1 male and 5 females, ranging from 30 to 44 years of age) living in an endemic region of disease (Belo Horizonte, Minas Gerais, Brazil). PBMCs were purified by density centrifugation through Ficoll-Hypaque (Sigma-Aldrich) as described by Oliveira-da-Silva *et al*. (2020). Cells (10^7^ per mL) were cultured in RPMI 1640 medium added with 20% FBS, 2 mm L-glutamine, 200 U mL^−1^ penicillin, 100 μg mL^−1^ streptomycin, 50 μM 2-mercaptoethanol, 1 mm sodium pyruvate and 1 × non-essential amino acid at pH 7.4. They were either left unstimulated (medium) or stimulated with rCHI or SLA (10 and 50 μg mL^−1^, respectively) for 120 h at 37°C in 5% CO_2_ incubator. Culture supernatant was collected and IFN-*γ* and IL-10 levels were measured using commercial kits (BD OptEIA™ Human IFN-*γ* and IL-10 ELISA Set, catalogues 555142 and 555157, respectively; BD Biosciences), as per the manufacturer's protocol.

### Proliferation induced by rCHI in splenocyte cultures after infection

Cell proliferation induced by rCHI was evaluated in spleen cell cultures after infection as described by Ratnapriya *et al*. (2021). Cells (10^6^ cells mL^−1^) were incubated in 100 μL of complete RPMI 1640 (Sigma-Aldrich) in 96-well flat-bottom tissue culture plates (Nunc, Denmark). They were either left unstimulated (medium) or stimulated with rCHI or SLA (10 μg mL^−1^, each) for 48 h at 37°C in 5% CO_2_ incubator. Concanavalin A (ConA, 5 μg mL^−1^; Sigma-Aldrich) was used as control. MTT 3-(4, 5-dimethylthiazol-2-yl)-2, 5-diphenyltetrazolium bromide (Sigma-Aldrich) was later added to the cultures and the OD values read in a Vmax microplate reader (Molecular Devices, USA) at 570 nm.

### *In vitro* toxicity induced by rCHI

The cytotoxic effect of rCHI was *in vitro* assessed in peritoneal macrophages obtained from naive mice. Cells were stimulated with 3% Fluid Thioglycollate Medium for 96 h at 37°C, when they were collected and plated (5 × 10^5^ cells per well) in 100 μl of complete RPMI in 96-well cell culture plates (Nunc). They were either left untreated (medium) or stimulated in triplicate with 1.0, 10.0 and 100.0 μg mL^−1^ of rCHI, SLA or AmpB. Cell viability was assessed through MTT assay after incubation for 48 h at 37°C in 5% CO_2_ incubator. The OD values were measured in a Vmax microplate reader (Molecular Devices) at 570 nm, and the cell viability was calculated by comparing the percentage of untreated and treated cells through the formula: cell viability(%) = (absorbance of treated cells/absorbance of untreated cells) × 100.

### Statistical analysis

Results were entered into Microsoft Excel (version 10.0) spreadsheets and analysed using GraphPad Prism™ (version 6.0 for Windows). One-way ANOVA followed by Bonferroni's post-test was performed to evaluate statistically significant differences between the experimental groups. Experiments were repeated twice, and results are representative of one of them. Differences were considered significant when *P* < 0.05.

## Results

### Construction and characterization of rCHI protein

**Figure 1.**
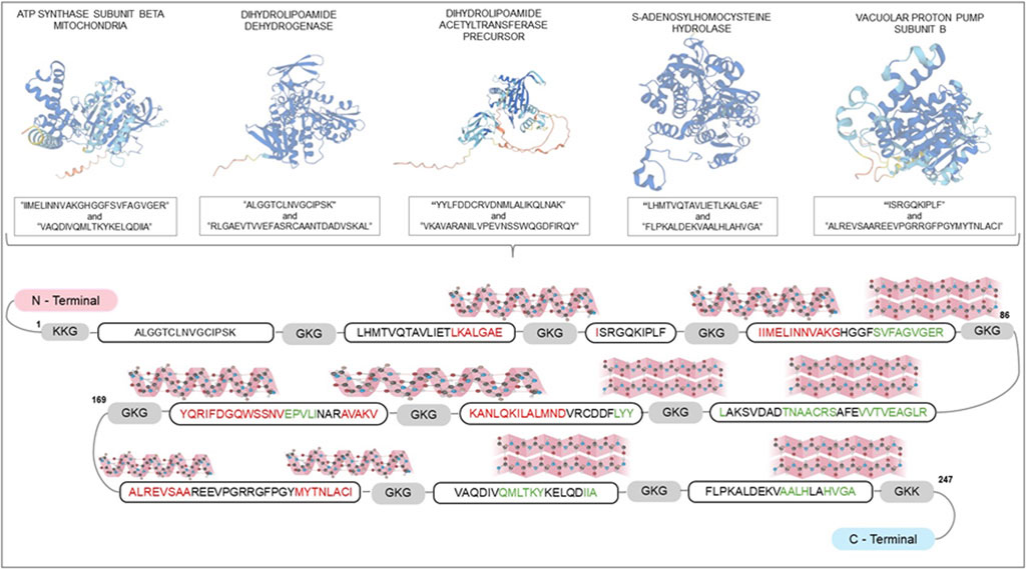
Alignment of T-cell epitopes used for the construction of the chimeric protein. Ten T-cell epitopes predicted from amino acid sequences of SUZ42449.1, AYU81901.1, SUZ46653.1, AYU83765.1 and AYU80416.1 proteins were linearly grouped with a spacing of 2 glycine and 1 lysine residues between them. The 3-dimensional structure provided by Uniprot server is presented in the first upper part of the figure. At the bottom, the protein is linearly showed with details of its secondary structure, which was provided by the jPred server, indicating the presence of alpha helices and beta sheets.

The amino acid sequences from 5 *Leishmania* proteins called ATP synthase subunit beta mitochondria (SUZ42449.1), dihydrolipoamide dehydrogenase (AYU81901.1), dihydrolipoamide acetyltransferase precursor (SUZ46653.1), S-adenosylhomocysteine hydrolase (AYU83765.1) and vacuolar proton pump subunit B (AYU80416.1) were evaluated to select human and murine CD4^+^ and CD8^+^ T-cell epitopes, which were used to construct the gene codifying rCHI. The following T-cell epitopes were predicted: ALGGTCLNVGCIPSK and RLGAEVTVVEFASRCAANTDADVSKAL of AYU81901.1; LHMTVQTAVLIETLKALGAE and FLPKALDEKVAALHLAHVGA of AYU83765.1; ISRGQKIPLF and ALREVSAAREEVPGRRGFPGYMYTNLACI of AYU80416.1; IIMELINNVAKGHGGFSVFAGVGER and VAQDIVQMLTKYKELQDIIA of SUZ42449.1; and YYLFDDCRVDNMLALIKQLNAK and VKAVARANILVPEVNSSWQGDFIRQY of SUZ46653.1 protein. They were grouped linearly with a spacing of 2 GLY and 1 LYS residues between them, and 2 additional LYS residues were added in the initial and terminal portions of protein sequence to make it soluble and easy to purify (Fig. 1). The Jpred 4 program was used to predict the protein secondary structure, and regions indicating alpha helices are highlighted in red, while beta sheets are indicated in green. The chimeric protein presents 247 amino acid residues, an estimated molecular weight of 26.4 kDa, and isoelectric point of 9.67. In addition, the instability index, aliphatic index and GRAVY values of rCHI are 17.36, 94.41 and −0.017, respectively.

### Cellular response developed before and after *L. infantum* infection

**Figure 2.**
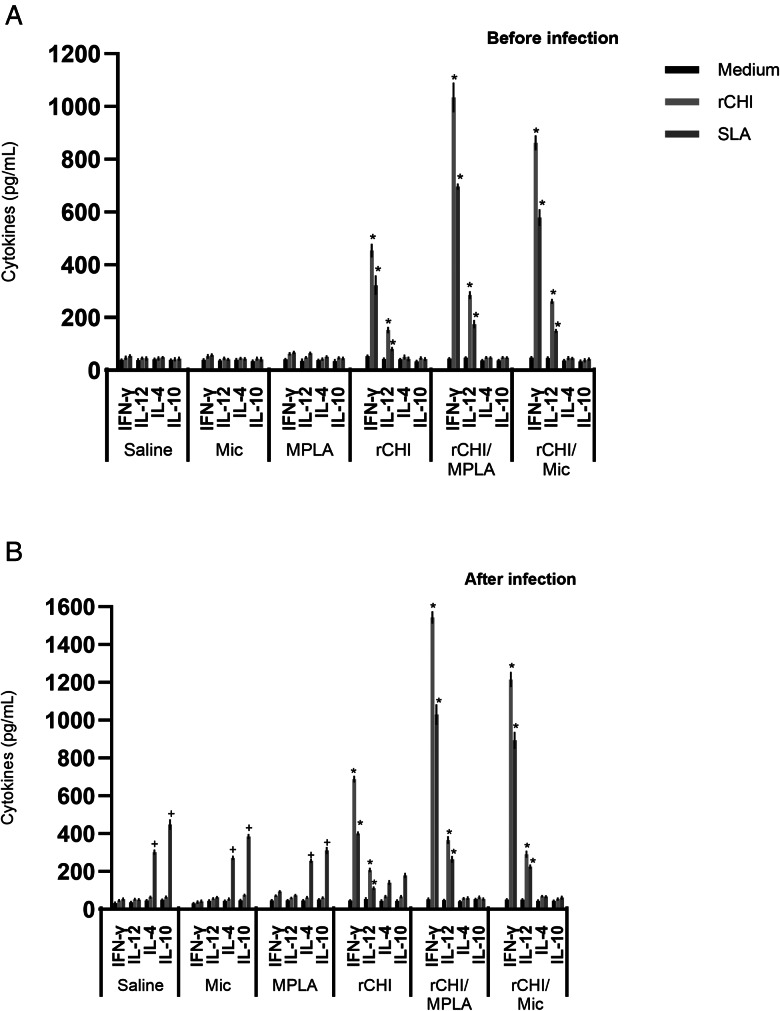
Cytokine production before and after *L. infantum* infection. Mice (*n* = 16 per group) received saline or were immunized with MPLA, micelles (Mic), rCHI, rCHI/MPLA or rCHI/Mic. Thirty days after the last vaccine dose, animals (*n* = 8 per group) were euthanized and spleens were collected. Splenocytes (5 × 10^6^ per well) were cultured in DMEM either left unstimulated (medium) or stimulated with rCHI or SLA (10 and 25 μg mL^−1^, respectively), for 48 h at 37°C in 5% CO_2_ incubator. Culture supernatant was collected and levels of IFN-*γ*, IL-12, IL-4 and IL-10 were measured through a capture ELISA (A). The others (*n* = 8 per group) were infected and, 45 days post-challenge, they were euthanized and spleens were collected and used in the same procedure. Results obtained in the cytokine levels are shown (B). Bars indicate the mean ± standard deviation of the groups. One-way ANOVA followed by Bonferroni's post-test was performed to evaluate statistically significant differences. (*) indicates significant difference in relation to the saline, Mic and MPLA groups (*P* < 0.0001). (^+^) indicates statistically significant difference in relation to the rCHI, rCHI/Mic and rCHI/MPLA groups (*P* < 0.01).

**Figure 3.**
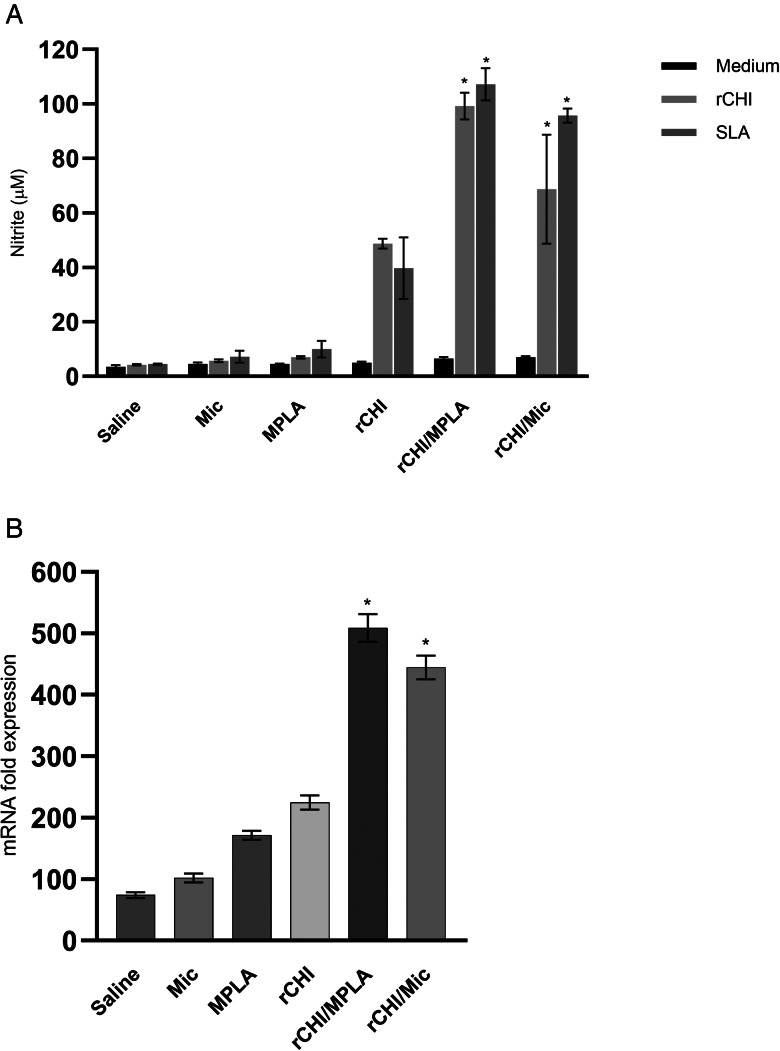
Nitrite production and IFN-*γ* mRNA expression after infection. Mice spleens (*n* = 8 per group) were collected on the 45th day after infection, and splenocytes (5 × 10^6^ per well) were cultured in DMEM and either left unstimulated (medium) or stimulated with rCHI or SLA (10 and 25 μg mL^−1^, respectively), for 48 h at 37°C in 5% CO_2_ incubator. Culture supernatant was collected and used to evaluate the rCHI- and SLA-specific nitrite production by Griess reaction (in A). In addition, stimulated cell cultures were used to extract RNA content, which was employed to evaluate the IFN-*γ* mRNA expression through RT-qPCR (in B). Relative gene expression is shown as 2^ − ΔΔCT. Bars indicate the mean ± standard deviation of the groups. One-way ANOVA followed by Bonferroni's post-test was performed to evaluate statistically significant differences. (*) indicates significant difference in relation to the saline, Mic, MPLA and rCHI groups (*P* < 0.0001).

**Figure 4.**
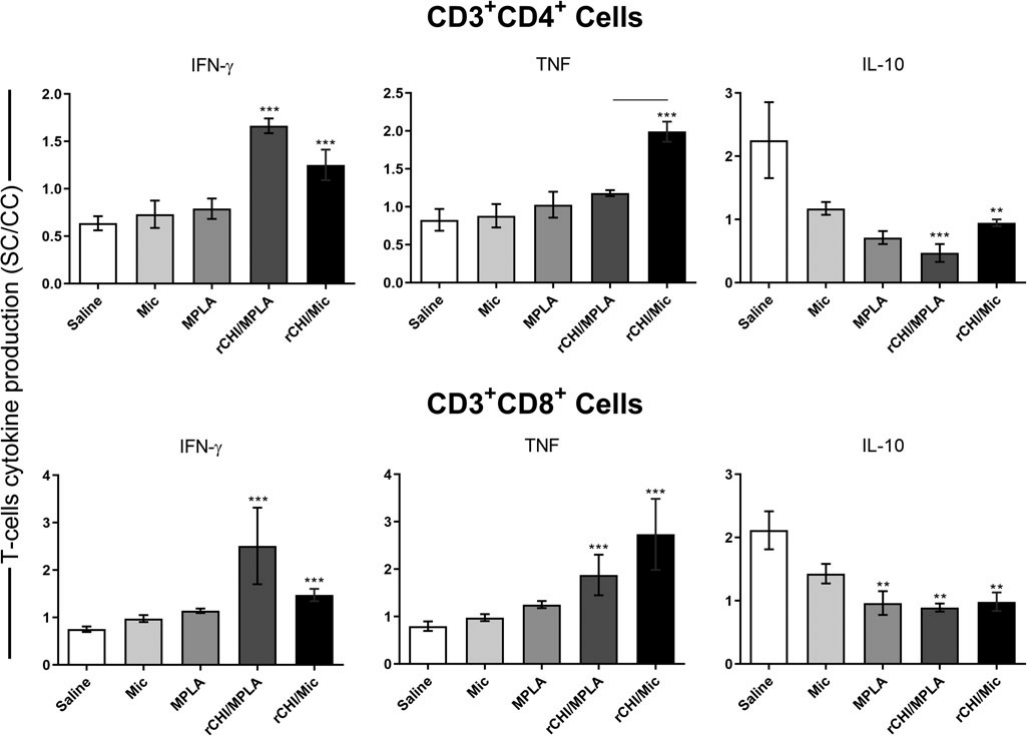
Flow cytometry analysis of T cells using spleen of *L. infantum*-infected mice. BALB/c mice were immunized and later challenged with *L. infantum* promastigotes. Forty-five days post-infection, spleens were collected and spleen cells were either left unstimulated (control) or stimulated with SLA (25 μg mL^−1^) for 48 h at 37°C in 5% CO_2_ incubator. The frequency (in terms of percentage) of T-cell producers of cytokines was obtained and values were used to calculate ratios between SLA-stimulated (SC) *vs* unstimulated (control, CC) cell cultures, which were presented as indexes. Bars indicate the mean ± standard deviation of the groups. One-way ANOVA followed by Bonferroni's post-test was performed to evaluate statistically significant differences. (**) indicates significant difference in relation to the saline and Mic groups. (***) indicates significant difference in relation to the saline, Mic and MPLA groups. The connecting line indicates significant difference (*P* < 0.05) between the rCHI/MPLA and rCHI/Mic groups.

We investigated if the rCHI vaccine, when associated with MPLA or micelles as adjuvant and/or delivery, could stimulate the development of a specific immune response in vaccinated mice. We used unvaccinated mice and those receiving only MPLA, micelle or protein as controls. Results showed that vaccination with rCHI/MPLA or rCHI/Mic induced significantly higher levels of IFN-*γ* and IL-12 in stimulated cell cultures, which were associated with significantly lower production of IL-4 and IL-10 before challenge (Fig. 2A). After infection, the cytokine profile was maintained unaltered in these animals. On the other hand, mice from control groups produced significantly higher levels of anti-SLA IL-4 and IL-10 cytokines, when compared to those vaccinated with rCHI plus MPLA or micelles (Fig. 2B). The nitrite production was evaluated as a marker of the macrophage's activation after infection, and results showed that rCHI/MPLA and rCHI/Mic induced significantly higher levels of antileishmanial nitrite, when compared to data obtained in control mice (Fig. 3A). The expression of IFN-*γ* mRNA in stimulated cell cultures was also evaluated after challenge, and results were significantly higher in the rCHI/MPLA and rCHI/Mic groups, as compared to the controls (Fig. 3B). The frequency of T cells producing cytokine was evaluated and results were presented as indexes derived from the ratio between the values found in the stimulated *vs* unstimulated cell cultures. Results showed that mice receiving rCHI/MPLA and rCHI/Mic and challenged presented higher ratios in the levels of IFN-*γ* and TNF-*α* producing CD4^+^ and CD8^+^ T cells, when compared to values found in control group mice, which showed higher ratios of IL-10-producing T cells (Fig. 4). We can then suggest that rCHI/MPLA and rCHI/Mic induced the development of a polarized Th1-type cellular response in mice from rCHI/MPLA and rCHI/Mic groups, as compared to the others, both before and after infection.

### Antibody production before and after infection

**Figure 5.**
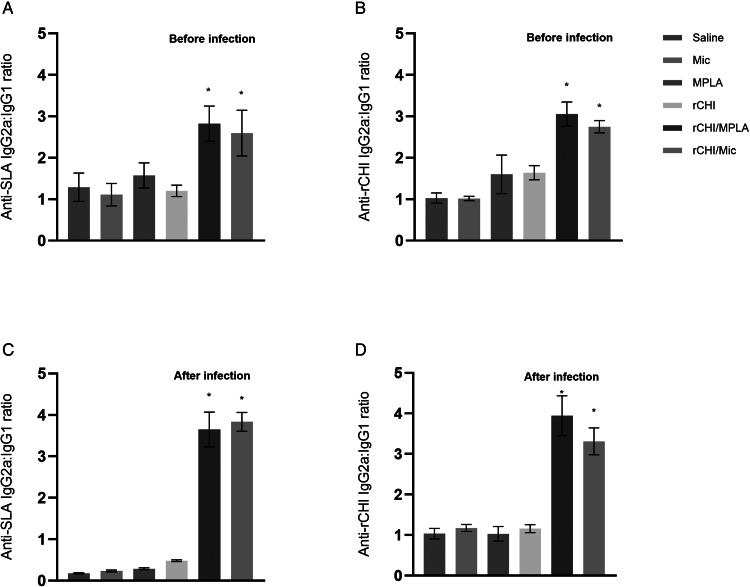
Humoral response before and after *L. infantum* infection. Mice (*n* = 16 per group) received saline or were immunized with MPLA, micelles (Mic), rCHI, rCHI/MPLA or rCHI/Mic. Thirty days after the last vaccine dose, blood samples from 8 animals per group were collected. The other mice (*n* = 8 per group) were infected and, 45 days post-challenge, their blood samples were collected. In both cases, blood was used to obtain serum and quantify the levels of anti-SLA and anti-rCHI IgG1 and IgG2a antibodies. The optical density (OD) values were obtained and used to calculate the ratios between the IgG2a/IgG1 levels, before (A and B) and after (C and D) challenge infection. One-way ANOVA followed by Bonferroni's post-test was performed to evaluate statistically significant differences. Bars indicate the mean ± standard deviation of the groups. (*) indicates significant difference in relation to the saline, Mic, MPLA and rCHI groups (*P* < 0.0001).

The humoral response was evaluated before and after infection by means of estimation of the levels of anti-rCHI and anti-SLA IgG1 and IgG2a antibodies. Before challenge, mice vaccinated with rCHI/MPLA and rCHI/Mic produced significantly higher levels of IgG2a antibodies, and ratios calculated using the OD values of both isotypes showed that these animals presented higher IgG2a/IgG1 levels, as compared to the other groups, by using SLA (Fig. 5A) or rCHI (Fig. 5B) as antigens. After infection, the humoral profile was maintained, since mice receiving rCHI/MPLA and rCHI/Mic produced higher levels of anti-rCHI and anti-SLA IgG2a antibodies, which reflected in higher ratios between IgG2a/IgG1 levels, as compared to the control groups, by using SLA (Fig. 5C) or rCHI (Fig. 5D) as antigens. In this context, rCHI/MPLA and rCHI/Mic group mice developed a specific Th1-type humoral response before and after infection.

### *In vivo* toxicity evaluated after *L. infantum* infection

**Figure 6.**
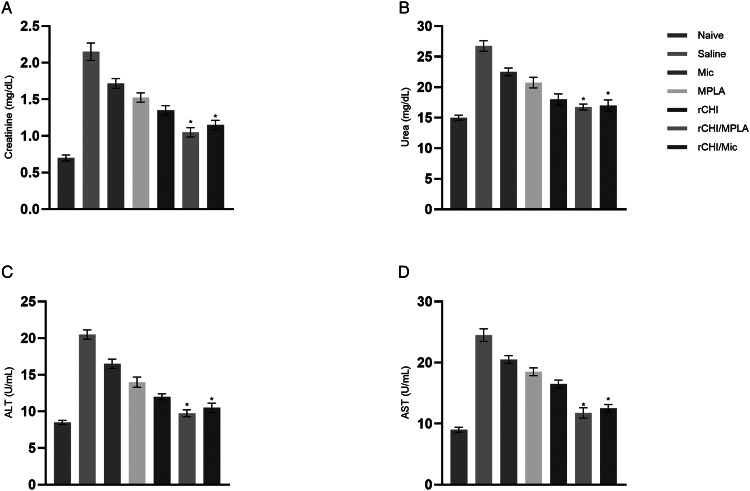
*In vivo* toxicity after *L. infantum* infection. The organic toxicity was evaluated in sera samples collected from infected animals (*n* = 8 per group). Levels of creatinine, urea, alanine aminotransferase (ALT) and aspartate aminotransferase (AST) were measured using commercial kits. Samples collected from non-infected and non-vaccinated (naive) mice were used as control. One-way ANOVA followed by Bonferroni's post-test was performed to evaluate statistically significant differences. Bars indicate the mean ± standard deviation of the groups. (*) indicates significant difference in relation to the saline, Mic, MPLA and rCHI groups (*P* < 0.0001).

We evaluated the organic toxicity after challenge infection, and results showed that the vaccination with rCHI/MPLA or rCHI/Mic reduced the levels of creatinine (Fig. 6A), urea (Fig. 6B), ALT (Fig. 6C) and AST (Fig. 6D), when compared to values found in control group mice, which presented higher levels of these renal and hepatic damage markers, suggesting possibly organic alterations caused by own *L. infantum* infection.

### Evaluation of the parasite load in infected and vaccinated mice

**Figure 7.**
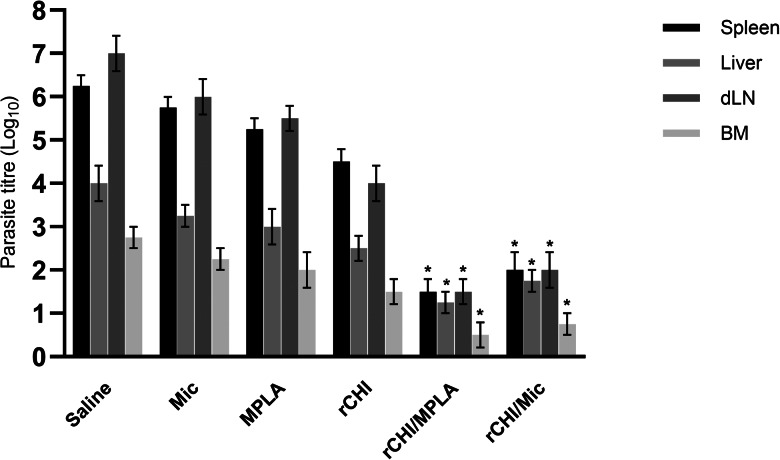
Parasite burden estimated through a limiting dilution assay. Mice (*n* = 8 per group) received saline or were immunized with MPLA, micelles (Mic), rCHI, rCHI/MPLA or rCHI/Mic. Thirty days after the last dose, they were challenged with *L. infantum* promastigotes; and 45 days post-challenge, spleens, livers, bone marrows (BMs) and draining lymph nodes (dLNs) were used to evaluate the parasite load through limiting dilution assay. Results were expressed as the negative log of the titre adjusted per milligram of organ. One-way ANOVA followed by Bonferroni's post-test was performed to evaluate statistically significant differences. Bars indicate the mean ± standard deviation of the groups. (*) indicates significant difference in relation to the saline, Mic, MPLA and rCHI groups (*P* < 0.0001).

**Figure 8.**
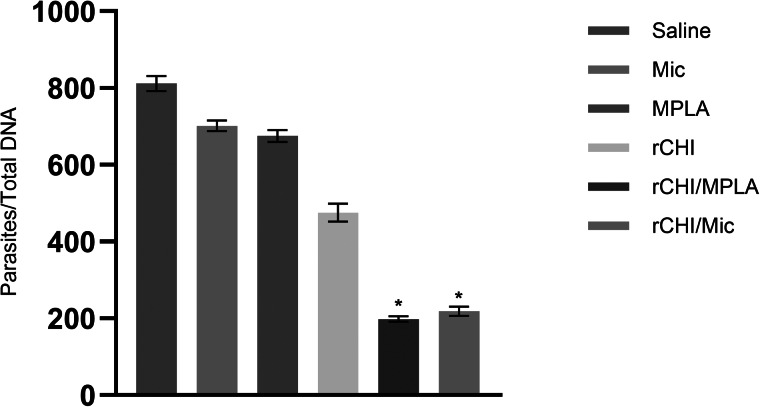
Splenic parasitism evaluated through qPCR. Mice (*n* = 8 per group) received saline or were immunized with MPLA, micelles (Mic), rCHI, rCHI/MPLA or rCHI/Mic. Thirty days after the last vaccine dose, they were challenged with *L. infantum* promastigotes; and 45 days post-challenge, spleens were used to evaluate the parasite load through qPCR. One-way ANOVA followed by Bonferroni's post-test was performed to evaluate statistically significant differences. Bars indicate the mean ± standard deviation of the groups. (*) indicates significant difference in relation to the saline, Mic, MPLA and rCHI groups (*P* < 0.0001).

The parasite load in animals’ organs was evaluated by a limiting dilution assay, and results showed rCHI/MPLA and rCHI/Mic induced significantly lower levels of parasitism in spleens, livers, BMs and dLNs of the animals, when compared to control mice (Fig. 7). In this context, reductions in the order of 4.8-, 2.8-, 2.3- and 5.5-log were found in livers, spleens, BMs and dLNs of rCHI/MPLA group mice, as compared to saline group, as well as reductions in the order of 4.3-, 2.2-, 2.0- and 5.0-log were found in livers, spleens, BMs and dLNs of rCHI/Mic group mice, as compared to data found in saline group mice. The splenic parasitism was also evaluated by qPCR, and results corroborated with those obtained by limiting dilution assay, with rCHI/MPLA and rCHI/Mic group mice presenting significant reductions in the order of 76.0 and 73.0%, respectively, in the splenic parasitism, as compared to values obtained in saline group mice (Fig. 8).

### *In vitro* immunogenicity induced by rCHI in human cells

**Figure 9.**
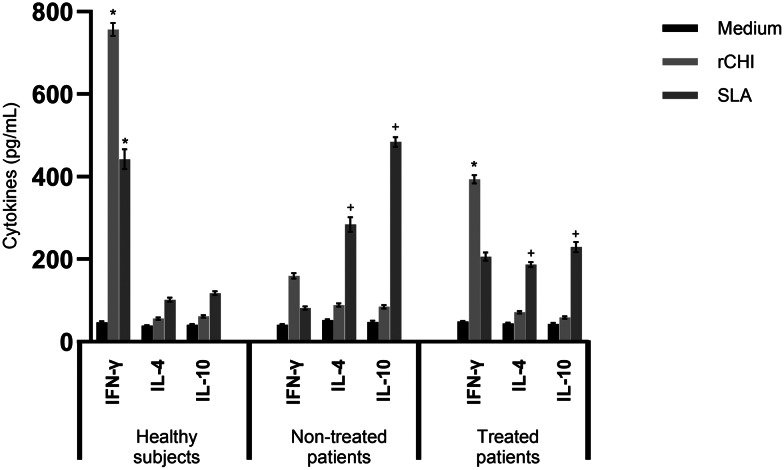
Immunogenicity induced by rCHI and SLA in human cell cultures. PBMCs (10^7^ cells per well) obtained from healthy subjects (*n* = 6) and VL patients (*n* = 6), those collected before and 6 months after their treatment, were either left unstimulated (medium) or stimulated with rCHI or SLA (10 and 25 μg mL^−1^, respectively) for 5 days at 37°C in 5% CO_2_ incubator. Culture supernatant was collected and IFN-*γ*, IL-4 and IL-10 levels were measured through a capture ELISA. One-way ANOVA followed by Bonferroni's post-test was performed to evaluate statistically significant differences. Bars indicate the mean ± standard deviation of the groups. (*) indicates statistically significant difference in relation to the unstimulated control (medium) or after SLA stimulus (*P* < 0.0001). (^+^) indicates statistically significant difference in relation to the stimulus using the rCHI protein (*P* < 0.0001).

The *in vitro* immunogenicity was also evaluated in PBMC culture obtained from VL patients before and after their treatment, as well as from healthy subjects. Results showed significantly higher levels of IFN-γ after rCHI stimulus, in both cell cultures from treated patients and healthy subjects, when compared to values obtained in untreated patients (Fig. 9). Otherwise, significantly higher levels of IL-4 and IL-10 were found when SLA was used as a stimulus of cell culture of untreated patients, as compared to values obtained after stimulation using rCHI.

### *In vitro* lymphoproliferation and toxicity in murine cells

**Figure 10.**
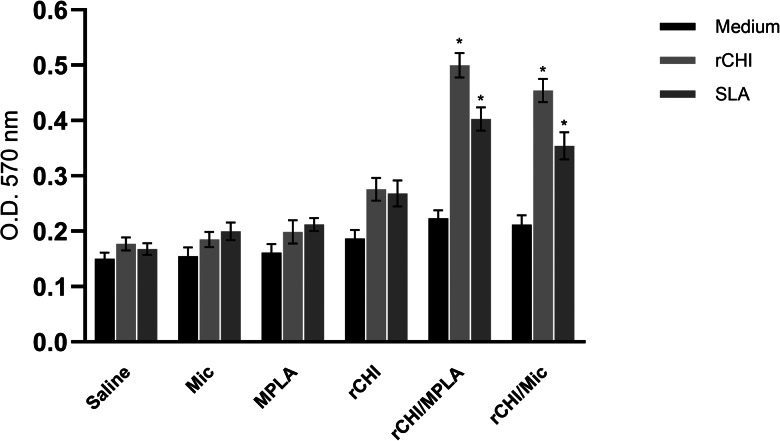
*In vitro* proliferative response induced by rCHI and SLA stimulation. The *in vitro* cell proliferation was evaluated in spleen cell cultures of infected animals (*n* = 8, per group). Cells (10^6^ per well) were incubated in complete RPMI 1640 medium and either left unstimulated (medium) or stimulated with rCHI or SLA (10 μg mL^−1^, each) for 48 h at 37°C in 5% CO_2_ incubator. MTT was added to the cultures and the OD values read in a microplate reader at 570 nm. One-way ANOVA followed by Bonferroni's post-test was performed to evaluate statistically significant differences. Bars indicate the mean ± standard deviation of the groups. (*) indicates significant difference in relation to the saline, Mic, MPLA and rCHI groups (*P* < 0.0001).

The *in vitro* proliferative response was evaluated in splenocyte cultures after infection, and results showed higher lymphoproliferation in cell cultures obtained from rCHI/Mic and rCHI/MPLA group mice, when compared to values found in control group mice, after stimulus using either rCHI or SLA (Fig. 10). Additionally, an *in vitro* toxicity study was performed using murine macrophages from uninfected and unvaccinated mice, and results showed that, even when a high concentration of rCHI or SLA was added in the cultures (100 μg well^−1^), a very low toxicity was found, with values of cellular viability higher than 92.0%. On the other hand, using AmpB at a concentration of 10 μg mL^−1^ results in the cellular viability of 38.5%.

## Discussion

Vaccination is a preventive measure considered against VL, which presents 2 main objectives, i.e. induction of a specific immune response to combat the parasite and/or prevent the infection, and the generation of long-lasting immunity (Abdellahi *et al*., 2022). Despite vaccine development advancements, few candidates are available to protect against canine VL as described by Duarte *et al*. (2016) and García-Castro *et al*. (2022), and a human vaccine still does not exist (Srivastava *et al*., 2016; Ikeogu *et al*., 2020). In this highlight, the search to reach an effective vaccine candidate continues. In the last decades, vaccination using single parasite proteins has allowed to the identification of candidates to protect against VL (Sachdeva *et al*., 2009; Coler *et al*., 2015; Machado *et al*., 2022a). However, this vaccine strategy presents drawbacks, since the concept of a single antigen to protect against a complex parasite has limitations, mainly related to the large antigenic repertoire of *Leishmania* spp. (Ostolin *et al*., 2021). Therefore, the idea of using multiple proteins or chimeric antigens containing distinct T-cell epitopes as vaccine candidates has highlighted new perspectives, and promising results have been obtained (Lage *et al*., 2022; Agallou *et al*., 2023). A multivalent vaccine presents benefits, which include low cost to manufacture and scalability, as well as the possibility to group distinct T-cell epitopes from different parasite proteins in a single molecule; which could be more effective to protect dogs and humans (Lage *et al*., 2020; Bhattacharjee *et al*., 2023).

In the present study, we developed a new multi-peptide vaccine based on the selection of 10 human and murine T-cell epitopes from 5 *L. infantum* proteins, and to use it to protect mice against VL. Results showed that rCHI induced cell proliferation in murine macrophage, with the association of higher production of IL-12 and lower levels of IL-4 and IL-10 obtained in the culture supernatants. Additionally, rCHI stimulated the development of a Th1-type cellular response in PBMC cultures from healthy subjects and treated patients, suggesting the possibility to be used as a human vaccine (Dias *et al*., 2018; Lage *et al*., 2020). We then prepared 2 compositions, i.e. rCHI/MPLA and rCHI/Mic, and tested them to immunize BALB/c mice. Results showed that both compositions stimulated the Th1-type response based on significantly higher levels of IFN-*γ*, TNF-*α* and IL-12 in stimulated splenocyte cultures; contributing to activate the microbicidal mechanisms of host cell to produce nitric oxide and other molecules to kill internalized parasites (Vakili *et al*., 2020; Elmahallawy *et al*., 2021).

In our study, rCHI alone induced some IFN-*γ* production and a slight degree of reduction of the parasite load in vaccinated animals; however, it was not sufficient to completely protect them from *L. infantum* infection. On the other hand, the association with MPLA or micelles increased the immunogenic effect of rCHI to the host immune system, making then possible to infer that the protection was improved by the association of such products with the recombinant antigen to protect against challenge infection (Das *et al*., 2018; Machado *et al*., 2022a). In fact, recombinant proteins usually require the association of adjuvants to induction of a stronger cellular response, with this activation based on CD4^+^ and CD8^+^ T cells. Thus, the vaccination using antigen and adjuvant can be considered as a requirement for better effectiveness of vaccine candidates to offer a complete protection against VL as described by others (Ratnapriya *et al*., 2019). MPLA is a Toll-like receptor-activating immune agonist that stimulates innate immunity resulting in the induction of an antigen-specific adaptive immunity. This adjuvant is approved for human use and it has been evaluated in clinical phase III trials (Van Haren *et al*., 2016; Li *et al*., 2023). Micelles composed of amphiphilic block copolymers have been also evaluated with therapeutic and prophylactic purposes. A variety of these polymers is evaluated as delivery and adjuvant in nanomedicine, and significant advances were made with the association of micelles to optimize the action of prophylactic and therapeutic candidates (Cabral and Kataoka, 2014). Here, we found that vaccination with the rCHI/MPLA and rCHI/Mic induced significant protection against *L. infantum* infection, since significant reductions in the parasite load were found in several organs of the animals, at 45 days post-challenge. Similar results were also found in other studies, where chimeric proteins plus adjuvants were employed to protect against murine VL (Lage *et al*., 2020; Agallou *et al*., 2023; Bhattacharjee *et al*., 2023); suggesting that the potential of our vaccine candidate to be protective against the disease.

Concomitantly to the evaluation of the parasitological and immunological efficacy of vaccine candidates; clinical safety assessments in mammals are also relevant with the purpose to verify if such candidates are or not toxic for the hosts, as well as if they can reduce the toxicity caused by their own *Leishmania* infection (Queiroz *et al*., 2022; Lage *et al*., 2023). In this context, toxicological studies such as those evaluating biochemical markers can contribute to indicate possible organic alterations in the infected animals (Mendonça *et al*., 2018). The dissemination of *L. infantum* to organs, such as BMs, livers and spleens cause alterations in the organic machinery and alter biochemical components in infected hosts (Carrión *et al*., 2006; Oliveira *et al*., 2012). This fact is consistent with the observation that spleen and BM seem to be sites of chronic infection, where parasites survive for long time (Rousseau *et al*., 2001; Lai *et al*., 2023). In our study, we found significantly lower levels of hepatic (ALT and AST) and renal (urea and creatinine) damage markers in mice from rCHI/MPLA and rCHI/Mic groups, as compared to values found in control mice. In fact, hepatocellular enzymes, such as AST and ALT, can be useful in detecting hepatic injury caused by damage in the cell membrane (Duarte *et al*., 2016). Otherwise, higher levels of urea and creatinine indicate alterations in the kidney function, which are commonly associated with alterations in the glomerular filtration rate (Torres *et al*., 2016). Therefore, we can conclude that both rCHI/MPLA and rCHI/Mic exerted a positive protective role in the animals’ organic function, contributing to reduced levels of hepatic and renal enzymes even in *L. infantum*-infected mice

The production of pro-inflammatory cytokines, such as IFN-*γ*, TNF-*α*, IL-12, among others, induces the nitric oxide production and oxygen-derivative molecules by host macrophages that are relevant for the *Leishmania* elimination (Vakili *et al*., 2020). Results showed that rCHI/MPLA and rCHI/Mic induced the production of high levels of these Th1-type cytokines in splenocyte cultures from vaccinated animals, with this high production being maintained after challenge. On the other hand, spleen cell cultures of control group mice produced higher levels of IL-4 and IL-10, indicating that the development of a Th2-type response and progression of the disease corroborated with the high parasitism found in such animals (Toepp and Petersen, 2020). These data support the potential protective role of rCHI plus adjuvant against VL. Similarly for the production of Th1-type cytokines, antibodies generated during infection are relevant to evaluate the susceptibility or resistance against infection. In fact, the induction of IgG1 and IgG2a isotypes corresponding to Th2- and Th1-type responses, respectively, contributes to the understanding of the immunological response developed in vaccinated hosts (Lehmann *et al*., 2000). Higher level of IgG2a is usually related to the presence of IFN-*γ*, while the production of IL-4 induces the switch of antibody isotype with the production of IgG1. In our study, significantly higher levels of anti-rCHI and anti-SLA IgG2a isotype antibodies were found after vaccination using rCHI/MPLA and rCHI/Mic, both before and after infection, suggesting the development of a Th1-type humoral response in such vaccinated animals.

The cytokine profile in PBMC cultures obtained from healthy subjects and VL patients was evaluated as a prospection for future use of rCHI as a human vaccine (Vale *et al*., 2019; Oliveira-da-Silva et al., 2020). The induction of cell-mediated immunity is important for the protection against human VL. In fact, VL stimulates the production profile of Th2-type cytokines, such as IL-4, IL-10 and TGF-*β*, as well as low proliferative response, when in *in vitro* cell cultures are stimulated with parasite antigens. In our study, rCHI stimulated the production of high levels of IFN-*γ*, when cell cultures from both healthy subjects and treated patients were stimulated with the protein. In addition, low levels of IL-4 and IL-10 were encountered in the culture supernatant, suggesting then that rCHI can active the human cells after *in vitro* stimulation. These data corroborate with others (Suman *et al*., 2018; Oliveira-da-Silva et al., 2020; Santos *et al*., 2021), in which recombinant proteins showed also stimulation of human immune cells for the production of Th1-type cytokines; thus altering the susceptibility profile associated with the active disease and Th2-type response.

Limitations of the study include the low number of samples from healthy subjects and VL patients, besides the absence of comparative measurements in antibody levels to corroborate with data obtained in the cytokine production. In addition, new experiments to evaluate the organic parasitism by means of histopathologic assays in infected animals should be also performed to align with data obtained in the parasite load by limiting dilution assay and qPCR. Future studies would also address the duration of protective response following the immunization with rCHI/MPLA and rCHI/Mic and challenge infection, with the purpose to determine the extension of the long-lasting Th1-type immunity in these animals. In this context, data presented here stand out to be considered as a proof-of-concept of this new recombinant antigen to induce immune stimulation in distinct mammalians and to protect against experimental VL.

## Supporting information

Lage et al. supplementary material 1 Lage et al. supplementary material

Lage et al. supplementary material 2 Lage et al. supplementary material

## Data Availability

All data are available in this article.

## Supplementary material

The supplementary material for this article can be found at https://doi.org/10.1017/S0031182024001240
