# Supplementary figures and images for: Preclinical evaluation of immunogenicity and protective efficacy of a recombinant chimeric protein vaccine against visceral leishmaniasis
Source: Parasitology. 2024 Nov 28;152(8):763–75. doi: 10.1017/S0031182024001240 (PMC12644939; doi:10.1017/S0031182024001240)

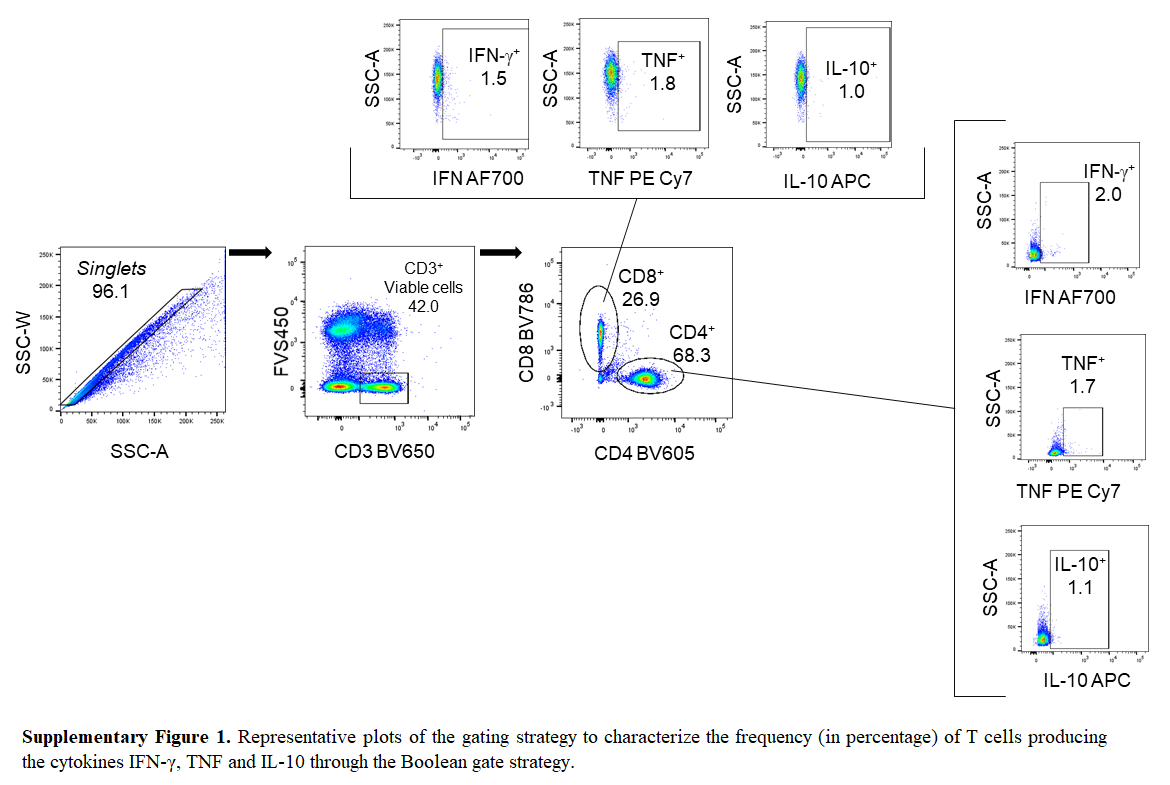

Supplement: Lage et al. supplementary material 1 — Lage et al. supplementary material [file S0031182024001240sup001.tif]
