# Supplementary material for: Preclinical evaluation of immunogenicity and protective efficacy of a recombinant chimeric protein vaccine against visceral leishmaniasis
Source: Parasitology. 2024 Nov 28;152(8):763–75. doi: 10.1017/S0031182024001240 (PMC12644939; doi:10.1017/S0031182024001240)
Supplement: Lage et al. supplementary material 2 — Lage et al. supplementary material [file S0031182024001240sup002.docx]

**Supplementary Table 1.** List of information about the antibodies and commercial kits used in the study.

| Product name | Catalog | Company | Address | Clone | Dilution factor |
| --- | --- | --- | --- | --- | --- |
| Pierce™ Chromogenic Endotoxin Quant Kit | A39552S | Thermo Scientific^®^ | Waltham, MA 02451, USA |  |  |
| Poloxamer P407 | 16758 | Sigma-Aldrich^®^ | St. Louis, MO 63103, USA |  |  |
| BD OptEIA mouse IFN-γ ELISA set | 555138 | BD Biosciences^®^ | San Diego, CA 92121, USA |  |  |
| BD OptEIA mouse IL-4 ELISA set | 555232 | BD Biosciences^®^ | San Diego, CA 92121, USA |  |  |
| BD OptEIA mouse IL-10 ELISA set | 555213 | BD Biosciences^®^ | San Diego, CA 92121, USA |  |  |
| BD OptEIA mouse IL-12 ELISA set | 555256 | BD Biosciences^®^ | San Diego, CA 92121, USA |  |  |
| TRIzol reagent | 15596026 | Invitrogen^®^ | Carlsbad, CA 92008, USA |  |  |
| UltraPure™ DNase/RNase-Free Distilled Water | 10-977-015 | Invitrogen^®^ | Carlsbad, CA 92008, USA |  |  |
| DNAse I | 18068015 | Invitrogen^®^ | Carlsbad, CA 92008, USA |  |  |
| High-Capacity cDNA Reverse Transcription Kit | 4368814 | Thermo Scientific^®^ | Waltham, MA 02451, USA |  |  |
| PowerUp™ SYBR™ Green Master Mix for qPCR | A25741 | Thermo Scientific^®^ | Waltham, MA 02451, USA |  |  |
| Brefeldin A | B6542 | Sigma-Aldrich^®^ | St. Louis, MO 63103, USA |  |  |
| Phorbol 12-myristate 13-acetate | P8139 | Sigma-Aldrich^®^ | St. Louis, MO 63103, USA |  |  |
| Ionomycin | I0634 | Sigma-Aldrich^®^ | St. Louis, MO 63103, USA |  |  |
| BD Horizon Fixable Viability Stain 450 | 562247 | BD Biosciences^®^ | San Diego, CA 92121, USA |  |  |
| Rat anti-mouse IgG1 secondary antibody, peroxidase-conjugated antibody | SA1-35640 | Invitrogen^®^ | Carlsbad, CA 92008, USA |  |  |
| Rat anti-mouse IgG2a secondary antibody, peroxidase-conjugated antibody | SA1-35646 | Invitrogen^®^ | Carlsbad, CA 92008, USA |  |  |
| Wizard Genomic DNA Purification Kit | A1120 | Promega^®^ | Madison, WI 53711 USA |  |  |
| BD OptEIA™ Human IFN-γ ELISA Set | 555142 | BD Biosciences^®^ | San Diego, CA 92121, USA |  |  |
| BD OptEIA™ Human IL-10 ELISA Set | 555157 | BD Biosciences^®^ | San Diego, CA 92121, USA |  |  |
| Concanavalin A | 11028-71-0 | Sigma-Aldrich^®^ | St. Louis, MO 63103, USA |  |  |
| Ficoll-Hypaque | GE17-1440-02 | Sigma-Aldrich^®^ | St. Louis, MO 63103, USA |  |  |
| 3-(4,5-Dimethyl-2-thiazolyl)-2,5-diphenyl-2H-tetrazolium bromide | 1.11714 | Sigma-Aldrich^®^ | St. Louis, MO 63103, USA |  |  |
| Alanine transaminase | 108-4/30 | Labtest Diagnóstica^®^ | Lagoa Santa, Minas Gerais, Brazil |  |  |
| Aspartate transaminase | 109-4/30 | Labtest Diagnóstica^®^ | Lagoa Santa, Minas Gerais, Brazil |  |  |
| Urea | 27-500 | Labtest Diagnóstica^®^ | Lagoa Santa, Minas Gerais, Brazil |  |  |
| Creatinine | 96-300 | Labtest Diagnóstica^®^ | Lagoa Santa, Minas Gerais, Brazil |  |  |
| Alexa Fluor® 700 Rat Anti-Mouse IFN-γ | 557998 | BD Biosciences^®^ | San Diego, CA 92121, USA | XMG1.2 | 1:50 |
| PE-Cy™7 Rat Anti-Mouse TNF | 557644 | BD Biosciences^®^ | San Diego, CA 92121, USA | MP6-XT22 | 1:50 |
| APC Rat Anti-Mouse IL-10 | 554468 | BD Biosciences^®^ | San Diego, CA 92121, USA | JES5-16E3 | 1:20 |
| PE Rat Anti-Mouse IL-2 | 554428 | BD Biosciences^®^ | San Diego, CA 92121, USA | JES6-5H4 | 1:20 |
| BV650 Rat Anti-Mouse CD3 Molecular Complex antibody | 740530 | BD Biosciences^®^ | San Diego, CA 92121, USA | 17A2 | 1:100 |
| BV605 Rat Anti-Mouse CD4 | 563151 | BD Biosciences^®^ | San Diego, CA 92121, USA | RM4-5 | 1:100 |
| BV786 Rat Anti-Mouse CD8a | 563332 | BD Biosciences^®^ | San Diego, CA 92121, USA | 53-6.7 | 1:100 |
